# Single-Stage Quadruple Valve Replacement with Mechanical and Bioprosthetic Valves: An 8-Year Follow-up Study from India

**DOI:** 10.1055/a-2591-9608

**Published:** 2025-05-26

**Authors:** Akriti Gera, Amit Misri, Pankaj Bajpai, Rajesh Sharma, Anil Bhan

**Affiliations:** 1 Department of Pediatric Cardiology, Medanta Hospital, Gurugram, Haryana, India; 2 Department of Pediatric Cardiac ICU, Medanta Hospital, Gurugram, Haryana, India; 3 Department of Cardio-Thoracic and Vascular Surgery, Cardiac and Thoracic Surgery, Medanta Hospital, Gurugram, Haryana, India

**Keywords:** quadruple valve replacement, rheumatic carditis, TOF

## Abstract

**Background:**

Congenital heart disease, repaired or unrepaired, requires lifelong follow-up. Carcinoid disease, rheumatic heart disease, and infective endocarditis can damage all four cardiac valves. Multiple valve replacement has a poor outcome.

**Case Description:**

A 23-year-old male underwent single-stage quadruple valve (mechanical [mitral and aortic] and bioprosthetic [tricuspid and pulmonary]) replacement due to rheumatic carditis and surgical complications. Over 8 years of follow-up, he is asymptomatic, has no prosthetic valve-related complications, and has improved cardiac function.

**Conclusion:**

Single-stage quadruple valve replacement with mechanical and bioprosthetic valves has a good long-term outcome. To the best of our knowledge, this is the longest follow-up study from India.

## Introduction

The natural history of congenital heart disease (CHD), including those who undergo cardiac surgery, is continuously evolving. The progressive nature of unrepaired defects deteriorates their quality of life. Those repaired, whether completely or palliatively, remain at high risk for acquired afflictions such as infective endocarditis (IE) or complications secondary to cardiac surgery. Hence, lifelong follow-up is prudent. Three causes of quadruple valve involvement have been reported in the literature: carcinoid disease, rheumatic heart disease (RHD), and IE. Owing to the high risk of surgical mortality and long-term morbidities related to prosthetic valves, 1 quadruple valve replacement (QVR) is seldom performed.

We report an 8-year follow-up in an adult who underwent single-stage QVR with mechanical and bioprosthetic valves, due to damaged native mitral (MV) and aortic valves (AoV), secondary to RHD, and damaged tricuspid and pulmonary valves post-intracardiac repair (ICR) of Tetralogy of Fallot (TOF).

## Case Report

**Video 1** Preoperative echocardiography.


**Video 2** Predischarge echocardiography.


A 23-year-old male, resident of Australia, presented to us in July 2015 with complaints of progressive exertional dyspnea, in New York Heart Association Class III. He underwent cardiac surgery at the age of 6 years for correction of TOF (Ventricular Septal Defect [VSD] closure with transannular patch). The surgical discharge summary was not available. He was being regularly followed up with a cardiologist in his hometown. During his regularly scheduled visit there, he complained of progressive exertional dyspnea. His echocardiography showed worsening valvular regurgitation, likely due to rheumatic carditis. He was informed of the same, and medical management was started. However, even after rheumatic activity settled, severe valvular regurgitation persisted. Hence, he was advised to undergo cardiac surgery.

On examination, the heart rate was 112/minute and blood pressure was 98/58 mm Hg. Cardiovascular examination revealed a pansystolic murmur in the tricuspid and mitral area and an end-diastolic murmur in the aortic and pulmonary area. Echocardiography showed ( Video 1 ) VSD patch intact with no residual shunt. However, severe tricuspid regurgitation was present, secondary to chordal rupture (likely during ICR), with a tricuspid mean gradient of 7 mm Hg (flow-related). Due to the transannular patch, he had severe pulmonary regurgitation. His MV chordae were thickened and calcified, causing severe mitral regurgitation and mild mitral stenosis (mean gradient of 4 mm Hg). A thickened tri-leaflet AoV with central non-coaptation was causing severe aortic regurgitation. All cardiac chambers were dilated, and biventricular dysfunction was present with a left ventricular (LV) ejection fraction of 30% to 35%. ECG revealed atrial flutter with right bundle branch block (RBBB), and chest X ray (CXR) showed cardiomegaly.

Due to severe involvement of all four cardiac valves, the patient was advised to undergo single-stage surgery. The probability of repair/replacement and the need for lifelong anticoagulation in the latter were explained.

**Fig. 1:**
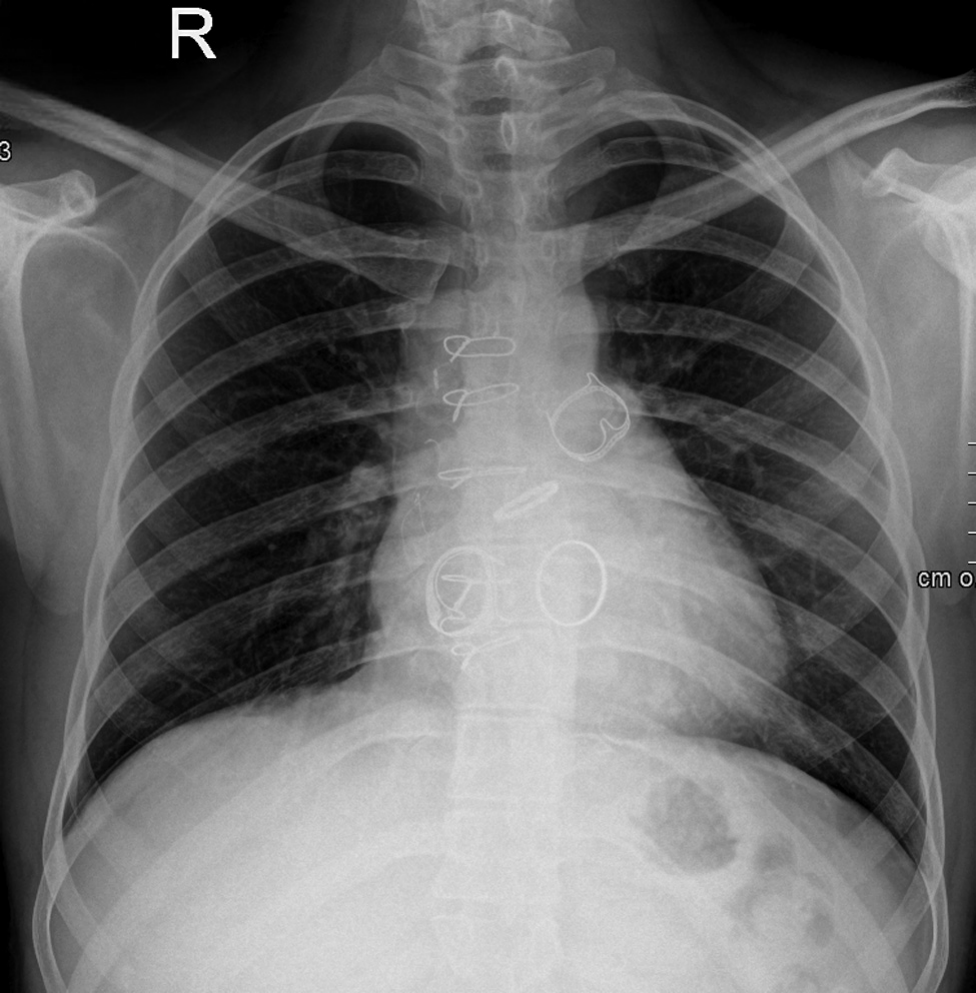
Post-surgery chest X-ray.

Our patient underwent single-stage quadrivalvular replacement on July 22, 2015. The surgery was performed with the patient on aorto-bicaval cardiopulmonary bypass via a midline sternotomy. Mitral and aortic valves were replaced by mechanical valves (27-mm St. Jude Medical [SJM] valve for MV and 21-mm SJM for AoV). Right-sided valves were replaced by bioprosthetic valves (tricuspid valve [TV] by 29 mm perimount valve and pulmonary valve [PV] by 25 mm perimount valve; Fig. 1 ).

The intraoperative period was uneventful. Postoperatively, we managed him with intravenous inotropes (epinephrine, norepinephrine, milrinone) and antiarrhythmics (intravenous amiodarone). He was extubated after 16 hours. Postoperative ECG showed RBBB with sinus rhythm. Postoperative echo revealed well-functioning prosthetic valves, Left Ventricular Ejection Fraction (LVEF) 35%, and no pericardial effusion. Anticoagulation was started on the second postoperative day with injection heparin, which was later substituted with warfarin after an adequate tide-over period, and the international normalized ratio (INR) was maintained between 2.5 and 3.

Histopathology of MV and AoV showed fibrinoid degeneration with leucocytic infiltrates, suggestive of rheumatic involvement. The histology of the tricuspid and pulmonary valves was normal.

His family was counselled regarding the importance of anticoagulation and its associated dietary and exercise restrictions. Predischarge echo revealed well-functioning prosthetic valves, prosthetic MV mean gradient was 6 mm Hg, prosthetic tricuspid valve mean was 6 mm Hg with mild tricuspid insufficiency, prosthetic AoV peak/mean gradient was 19/13 mm Hg with mild aortic regurgitation, prosthetic pulmonary valve peak/mean gradient was 11 mm Hg with mild pulmonary regurgitation, and mild biventricular dysfunction. He was discharged after a 15-day hospital stay on oral antihypertensives (ACE inhibitor), beta blockers, decongestants, penicillin, and anticoagulants (warfarin and aspirin). His target INR was advised between 2.5 and 3.

He was followed up for 1 week and 1 month after discharge. Good prosthetic valvular function with mild LV dysfunction was noted. Thereafter, he was advised to continue cardiac medications and warfarin. Due to international travel, his follow-up with us was sparse but regular.

At the 2-year post-surgery visit, INR levels were well maintained, and echocardiography showed all prosthetic valves were well functioning and LVEF had improved to 50%. Annual visits thereafter had similar echocardiography findings, and he was continued on ACE inhibitors, warfarin, and aspirin.

Our patient is now 31 years old and has been on regular follow-up for 8 years. His last visit with us was in April 2023. He was asymptomatic and compliant with medications (warfarin and aspirin) and maintaining an INR between 2.5 and 3. There was no history of thromboembolic or bleeding complications. On examination, he had stable vitals, and a valve click was present. Echocardiography revealed ( Video 2 ) VSD patch intact with no residual shunt, functioning mechanical prosthetic valves in mitral and aortic position; MV mean gradient of 7 mm Hg, no mitral regurgitation/stenosis, AoV peak gradient of 24 mm Hg, no aortic regurgitation/stenosis, functioning bioprosthetic prosthetic valves in tricuspid and pulmonary position; moderate tricuspid regurgitation with mean tricuspid gradient of 10 mm Hg, mild pulmonary insufficiency with peak gradient of 38 mm Hg, normal biventricular functions. We have advised him to continue his anticoagulation and maintain his INR as per target range, to continue injection penicillin and subacute bacterial endocarditis prophylaxis as per guidelines.

## Discussion

CHD and its repair affect our patients lifelong. If unrepaired, CHDs decrease life expectancy with every bout of complications. When we repair the structurally abnormal heart, long-term follow-ups are paramount to understand if we truly achieved the benefits we intended to.

Single or double valve replacements have been commonly performed, and patients do well on long-term follow-ups. There is overall hesitance in triple valve replacements due to reports of high operative mortality (up to 25%) and low 10-year survival rate (61%), despite improvements in operative and myocardial protection techniques. 2 Moreover, the replacement of multiple valves exposes the patient to added long-term morbidities related to prosthetic valves, such as thromboembolism, anticoagulation-related hemorrhage, endocarditis, and paravalvular leakage, compared with the replacement of single valves. 1 Patients need to be extremely compliant with warfarin, which is a difficult drug to manage because of its narrow and controversial therapeutic window and potentially serious side effects. Hence, it is understandable why QVR is uncommonly performed.

Very few cases of single-stage QVR have been reported in the literature. It has been performed secondary to rheumatic carditis, 3 4 IE, 5 6 7 8 and carcinoid disease. 9 10 Rheumatic carditis commonly affects the mitral or mitral and aortic valve. Tricuspid and pulmonary valve involvement is rare. In our patient, the etiology was an interesting mixture. Mitral and aortic valves were afflicted with rheumatic carditis, which led to severe regurgitation of both. But severe tricuspid and pulmonary regurgitation was secondary to the first cardiac operation. While the tricuspid valve appeared damaged secondary to iatrogenic chordal rupture, the pulmonary valve was severely insufficient due to a transannular patch, which is necessary in repairing a hypoplastic pulmonary annulus.

Follow-up studies of QVR are even fewer. 4 6 8 The earliest, and possibly first, follow-up study of QVR in literature is published by Hossack et al., 4 who in 1987 followed up their 60-year-old patient, 3.5 years after sequential QVR (triple valve replacement followed by tricuspid valve replacement) for rheumatic carditis. They reported decreased symptoms and improved hemodynamics.

Cao et al. 6 followed their patient for 11 years post-quadruple mechanical valve replacement (due to IE). Their patient remained asymptomatic throughout while maintaining target INR between 1.5 and 2, which is considerably lower than recommended, which they attributed to the lesser vulnerability of Asians to thrombotic diseases, compared to Caucasians.

In 2023, Wen et al. 8 reported improved clinical status follow-up findings 6 months post-single-stage QVR with mechanical valves in a patient who developed IE 16 years post-TOF repair.

The 2020 American College of Cardiology/American Heart Association (ACC/AHA) valvular heart disease guidelines 11 restrict mixed valvular diseases to those involving mitral and aortic only. Additionally, warfarin cannot be exempted if mechanical valves are used. Target INR in mechanical AoV replacement without risk factors is 2.5, which is increased to 3 in cases of associated risk factors (older generation valve, atrial fibrillation, previous thromboembolism, hypercoagulable state, and LV systolic dysfunction) or mechanical MV replacement. In cases of a bioprosthetic valve, aspirin is recommended. INR recommendations for right-sided valve replacements are a gray area. The 2021 European Society of Cardiology/European Association for Cardio-Thoracic Surgery (ESC/EACTS) guidelines 12 for management of valvular heart disease recommend intervening in mixed valvular diseases depending on the predominant lesion. Additionally, they recommend target INR based on thrombogenicity of the valve and patient risk factors (mitral or tricuspid valve replacement, previous thromboembolism, atrial fibrillation, mitral stenosis of any degree, LVEF <35%). We decided to target INR from 2.5 to 3 based on the mechanical AoV.

Our study is unique, albeit single. Our compliant patient has proven the long-term benefits of valve replacement in a setting of controlled INR monitoring. He has religiously maintained his INR between 2.5 and 3. He has not suffered any thromboembolic, bleeding, or structural valve deterioration complications. His sequential echoes showed improved cardiac function and hence has been in good health. To the best of our knowledge, this is the only study where all native valves were replaced using both bioprosthetic and mechanical artificial valves, in a single stage operation and with the longest follow up from India.
